# Visual system assessment for predicting a transition to psychosis

**DOI:** 10.1038/s41398-022-02111-9

**Published:** 2022-08-29

**Authors:** Alexander Diamond, Steven M. Silverstein, Brian P. Keane

**Affiliations:** 1 Department of Psychiatry, University of Rochester Medical Center, 601 Elmwood Ave, Rochester, NY USA; 2 Department of Neuroscience, University of Rochester Medical Center, 601 Elmwood Ave, Rochester, NY USA; 3 Center for Visual Science, University of Rochester, 601 Elmwood Ave, Rochester, NY USA; 4 Department of Ophthalmology, University of Rochester Medical Center, 601 Elmwood Ave, Rochester, NY USA; 5 Department of Brain & Cognitive Sciences, University of Rochester, 358 Meliora Hall, NY Rochester, USA

**Keywords:** Predictive markers, Schizophrenia, Diagnostic markers, Molecular neuroscience

## Abstract

The field of psychiatry is far from perfect in predicting which individuals will transition to a psychotic disorder. Here, we argue that visual system assessment can help in this regard. Such assessments have generated medium-to-large group differences with individuals prior to or near the first psychotic episode or have shown little influence of illness duration in larger samples of more chronic patients. For example, self-reported visual perceptual distortions—so-called visual basic symptoms—occur in up to 2/3rds of those with non-affective psychosis and have already longitudinally predicted an impending onset of schizophrenia. Possibly predictive psychophysical markers include enhanced contrast sensitivity, prolonged backward masking, muted collinear facilitation, reduced stereoscopic depth perception, impaired contour and shape integration, and spatially restricted exploratory eye movements. Promising brain-based markers include visual thalamo-cortical hyperconnectivity, decreased occipital gamma band power during visual detection (MEG), and reduced visually evoked occipital P1 amplitudes (EEG). Potentially predictive retinal markers include diminished cone a- and b-wave amplitudes and an attenuated photopic flicker response during electroretinography. The foregoing assessments are often well-described mechanistically, implying that their findings could readily shed light on the underlying pathophysiological changes that precede or accompany a transition to psychosis. The retinal and psychophysical assessments in particular are inexpensive, well-tolerated, easy to administer, and brief, with few inclusion/exclusion criteria. Therefore, across all major levels of analysis—from phenomenology to behavior to brain and retinal functioning—visual system assessment could complement and improve upon existing methods for predicting which individuals go on to develop a psychotic disorder.

## Introduction

Schizophrenia (SZ) cuts lifespan short by 20 years, leaves about 80% unemployed, and costs society over $100 billion annually [1, 2]. Despite the tremendous burden that the illness places on patients, family, and society at large, the field of psychiatry is still far from perfect in predicting who will end up transitioning to a psychotic disorder. Current practice is to employ instruments such as the Structured Interview for Psychosis Risk Syndromes (SIPS) and Comprehensive Assessment of At-Risk Mental States (CAARMS) to designate help-seeking individuals as being at clinical high risk for psychosis (CHR). However, because only 20-25% of those with this designation develop a psychotic disorder within 3 years from initial assessment, additional predictive measures are needed [3]. Improving our predictive tools could have real impact, first, because early intervention can potentially delay illness onset or improve clinical course [4, 5]; second, because many individuals designated as CHR will fully remit and not need long-term treatment [6]; and third, because early detection can reduce the number of emergency hospital visits and thereby minimize overall health care expenditures [7]. To provide more effective, targeted, and lower-cost treatment, what is needed are additional predictors for discerning who will ultimately transition to a psychotic disorder. We propose that visual system assessment can help in this regard.

Why focus on vision? Almost 30% of human cortex is devoted to visual functioning [8] and schizophrenia is increasingly regarded as a disorder that afflicts neurons throughout the entire brain [9, 10]. Thus if any brain system should distinguish the disorder, the visual system should, and there is already supporting evidence from large-scale resting-state functional connectivity studies [11]. Visual hallucinations occur in at least a quarter of those in the psychosis spectrum [12]. Visual disturbances—which often do not qualify as hallucinatory because they occur too infrequently or because they merely alter stimulus appearance—are reported by almost two-thirds of people with non-affective psychosis [13–15], which rivals the prevalence of more standard illness features such as auditory hallucinations [16].

We argue that phenomenological, behavioral, brain-based, and retinal functioning tests may all provide markers for predicting a transition to a psychotic disorder. We make this argument not by providing an exhaustive review of all candidate markers but by describing a small number of promising examples. The visual assessments that we highlight have either: (i) distinguished CHR individuals who convert from those who do not convert to a psychotic disorder, (ii) distinguished first-episode patients (FEPs) from well-matched controls, or, at the very least, (iii) distinguished more chronic schizophrenia patients (“SZs”) independently of illness duration and relative to a psychiatric control group. As described below, group differences generated by these selected assessments cannot be explained by medication or attentional/motivational impairments; they can be observed in clinically stable outpatients (hence being suitable for CHR populations); they have been shown relative to healthy and often mood disorder control groups (the most common final diagnosis in CHR populations [17]); and they generate medium-to-large effect sizes (Cohen’s d ranging from 0.5 to 1.4 corresponding to AUC values of 0.64–0.84 and odds ratios of 2.5–12.8) [18].

Visual system assessments were discovered through our past reviews [19–21] and more recent PubMed abstract/title literature searches in English (search date: 7/12/2022). Keywords used in the search were: (“at risk” OR “high risk” OR prodom* OR “attenuated psychosis” OR “first episode” OR “recent onset”) AND (vision OR visual OR occipit* OR retin*) AND (schizophrenia OR psychosis OR psychotic). Note that we do not report whether CHR populations as a whole differ from controls since roughly three-quarters of such individuals never develop a psychotic disorder [3] and conversion-specific effects would become diluted in such samples (e.g., *d* = 0.8→*d* = 0.2). In a similar vein, it is neither necessary nor sufficient for visual measures to generate distinctive results in first-degree relatives or in people with schizotypal features (or schizotypal personality disorder) compared to controls, since such individuals rarely develop a psychotic disorder. The candidate markers that we seek would ideally be akin to the Hopkins Verbal Learning Test-Revised of the NAPLS psychosis risk calculator [22]: they would signify a state that encompasses the period up through the first psychotic episode, but could additionally (and optionally) reflect trait vulnerability.

The remaining part of this article is structured as follows: first, we describe potential phenomenological, behavioral, brain-based, and retinal markers of early psychosis; then we argue that many of these markers could realistically be assayed in everyday clinical settings; next, we briefly speculate on how these visual measures might shed light on the mechanisms undergirding a transition to psychosis; and we conclude by addressing objections and limitations.

### Self-reported visual perceptual abnormalities: moving beyond hallucinations

While objective measures are obviously preferred, symptom-based measures cannot be ignored when they have already demonstrated their clinical utility. Seventeen non-hallucinatory changes to everyday visual perceptual experience are probed by the Bonn Scale for Assessment of Basic Symptoms [23]. The scale taps into lifetime distortion occurrence with a binary response format, freeing patients from having to remember the graded frequency, duration, timeframe, or life impact of the symptoms, all of which can be clouded by memory impairment or experimenter demand bias [24]. The subscale can be quickly administered (~20 min), and has good test-retest reliability and inter-rater reliability [15, 25]. The summed scores are elevated in ~2/3rds of unmedicated FEPs [15], and can cross-sectionally distinguish people with SZ and schizoaffective disorder from those with other psychotic disorders [13, 26]. Most importantly, in a longitudinal study with a 9.6 year follow-up, visual distortions could predict which psychiatric help-seeking individuals would go on to develop SZ (sensitivity = 0.46, specificity = 0.85, positive predictive value = 0.75, negative predictive value = 0.62) [27]. Unfortunately, instruments that label individuals as being at clinical high risk (SIPS/CAARMS) only briefly touch upon non-hallucinatory visual disturbances with 2–3 anchor questions. Because these disturbances are subtle, infrequent, and phenomenologically diverse—that is, because they are easily missed on routine interviews—asking the full set of visual questions from the Bonn Scale may be needed to optimize clinical prediction.

### From neuropsychology to visual psychophysics: broadening the scope of “cognition”

“Cognitive” deficits traditionally include those in the domain of working memory, executive functioning, knowledge acquisition, processing speed, and logical reasoning [28]. Neurocognitive tests can detect deficits nearly a decade before illness onset [29] and can improve predictions as to which CHR individuals will go on to develop a psychotic disorder [22]. Can these tests be improved upon? If so, how? Here, we make a simple recommendation, namely, to *expand the definition of “cognition”* to encompass aspects of visual perceptual functioning. We propose that a vision-science-based test battery, when combined with other clinical and neurocognitive tools, can boost the likelihood of detecting a psychotic disorder in addition to clarifying aspects of pathophysiology. This is so because methods of behavioral psychophysics have matured considerably over the last 150 years, and can precisely and efficiently reveal group differences in a way that can often be linked to underlying mechanisms [30]. A visual battery, if implemented, would be inexpensive, (mostly) objective, and strongly justified by past psychosis research.

**Fig. 1:**
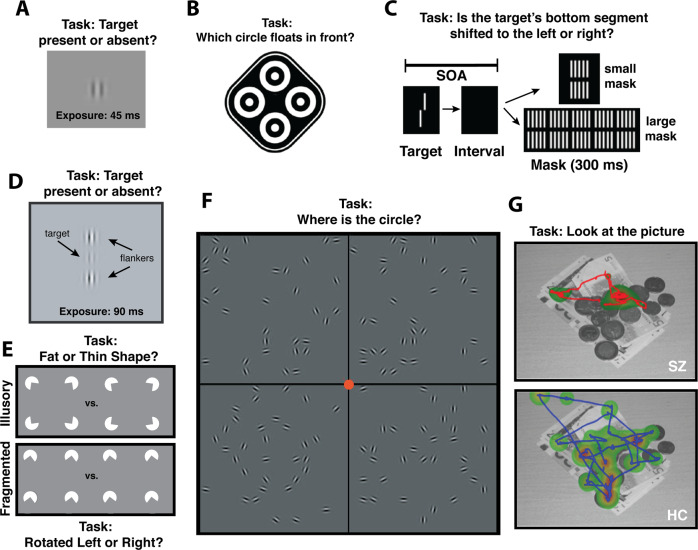
Visual tasks that may elicit behavioral markers of early psychosis. **A** Never-medicated first-episode psychosis patients (FEPs) have superior contrast sensitivity at lower spatial frequencies compared to healthy controls (HCs) [32]. **B** Never-medicated FEPs have a diminished ability to identify the circle that floats in stereo [35]. **C** FEPs need more of a temporal interval (SOA) between the target and a subsequent grid-like mask to achieve 75% accuracy on a Vernier discrimination task [42]. **D** Schizophrenia patients of varying illness durations benefit less from high-contrast collinear flankers when attempting to detect a central low-contrast target relative to healthy and clinical controls [45, 47]. **E** For FEPs, discriminating fat and thin illusory shapes is harder than discriminating left and right rotated pac-men; for HCs, the opposite is true [48]. **F** Compared to healthy controls, FEPs can tolerate fewer background “noise” elements when attempting to detect a circular chain of elements [50]. **G** Independently of illness duration, SZ patients exhibit spatially restricted eye movement patterns when freely viewing complex images [51].

But which tasks might be included? Here, we describe seven candidates (see Fig. 1). In a contrast sensitivity task, participants attempt to detect a briefly presented (≤300 ms), low-contrast target on a constant luminance background. Never-medicated FEPs express superior sensitivity (*d* = 1.19, *p* < 0.001, *n* = 20/group), especially for lower spatial frequency elements (≤5 cycles/deg) [31]. The perceptual advantage has been reported in a separate sample of unmedicated FEPs [32] and in unmedicated older patients using drifting gratings [33].

In a Titmus test (also referred to as the Wirt circles or graded circles test), participants wear polarized glasses and serially examine 9 arrays of four circles, indicating which one hovers in depth (Stereo Optical Company Inc, Chicago, IL). SZ patients and drug-naïve FEPs have shown a similarly reduced ability to identify the target across a broad range of stereo-disparities [34–36] (*d* ≥ 0.67).

**Fig. 2:**
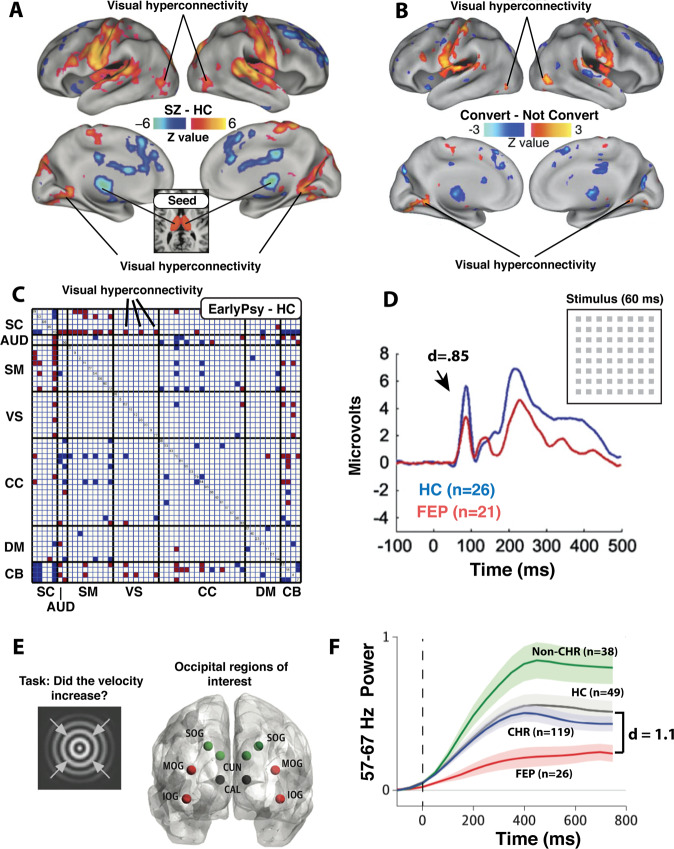
Visually oriented brain-based markers of early psychosis. **A** Visual thalamo-cortical hyperconnectivity occurs in schizophrenia patients relative to HCs (Figure panel shows the thalamic seed; adapted with permission from [58]). **B** Similar hyperconnectivity patterns have been observed in CHR patients who convert versus those who do not convert to a psychotic disorder (Adapated with permission from [61]). **C** Different investigators have found comparable patterns (after FDR correction) in early-stage non-affective psychosis patients relative to HCs, with one-third of the visual nodes being hyperconnected to the thalamic node (Adapted with permission from [60]). **D** Briefly flashed checkered images have elicited a muted P1 amplitude in FEPs relative to HCs (Adapted with permission from [65]). **E**, **F** During a change-in-motion detection task, gamma band activity within ten occipital regions was smallest in FEP subjects, intermediate in CHR patients, and greatest in psychiatric patients not meeting CHR criteria (non-CHR) (Adapted with permission from [68]).

When attempting to identify a briefly presented target, medicated and unmedicated schizophrenia patients are more adversely affected by a spatially overlapping distractor that appears within ~100 ms of target onset; this exaggerated “masking” happens relative to healthy controls and first-degree biological relatives [37, 38]. Such masking differences have also been observed in first-episode schizophrenia patients compared to healthy controls (179 HCs, 86 patients; *d* = 1.1, *p* < 0.001) and two groups at risk for psychosis (both *d* > 0.85; both *n* > 38, both *p* < 0.001) [39]. A limitation in this study was that masking was operationalized as raw accuracy in target detection, introducing the possibility of motivational/attentional confounds [39]. In a more recent “shine-through” version of the masking task, subjects determine the relative alignment of two line segments (vernier discrimination) that are then followed by a grid of segments (with small or large masks, Fig. 2) [40]. Shine-through studies have mitigated attentional confounds by excluding subjects who fail to achieve some minimal level of discrimination performance with briefly flashed unmasked stimuli. Schizophrenia patients have shown more shine-through masking than major depression patients [41] (34 depressed, 90 SZs, *d* > 0.8, *p* < 0.001) and FEPs have shown more shine-through masking than healthy controls (20 HCs, 21 FEP; *d* = 0.52, *p* = 0.02 without statistical correction) [42].

In a collinear facilitation task, a faint target becomes more detectable when flanked by collinear high-contrast elements [43]. Whereas healthy controls and people with bipolar I disorder benefit normally from collinear flankers, the same is not true for clinically stable SZ outpatients [44]. The facilitation impairment arises among SZ patients with intact attention, intact contrast sensitivity, and 12-week medication washout periods [45, 46]. In a sample of 38 SZ patients (including 6 FEPs), the deficit did not vary with illness duration [47]. A caveat is that the effect may be most apparent for elements that have a smaller Gaussian window (width) or higher spatial frequency, perhaps because such stimuli bias processing toward unmyelinated long-range horizontal connections in early visual cortex [47].

In a shape completion task, four pac-men form a fat or thin shape or are individually rotated leftward or rightward (illusory and fragmented condition, respectively). SZ patients show more deficits in the illusory than fragmented condition (134 non-affective psychosis patients, 66 HCs; *d* = 0.67, *p* < 10^–4^) [48]. These impairments are larger in schizophrenia than in bipolar disorder [49] and emerge maximally by the first psychotic episode (23 FEPs, 48 HCs; *p* < 0.01, *d* = 0.74) [48]. Note that because the shape completion impairment is defined as a within-subject difference (fragmented – illusory threshold), the group difference cannot be blamed on inadequate attention, motivation, visual acuity, or orientation discrimination.

FEPs are also impaired at locating chains of co-aligned edge elements embedded within varying amounts of randomly oriented “noise” elements [50]. This contour integration deficit, which becomes apparent for spatially-scaled down (smaller) displays (*d* = 0.72, *p* < 0.05), cannot be explained by inattentiveness since the same patients perform near ceiling on catch trials (where the target was shown by itself) [50].

Eye movement differences also hold promise. Using a dot fixation and free-viewing task, a probabilistic neural network classifier could distinguish healthy controls (*n* = 148) and clinically stable SZ patients (*n* = 150) with 94% accuracy. The group differences did not depend on illness duration, anxiety, symptom severity, caffeine/nicotine use, or medication [51]. The group differences were stable over a 9 month period, generalizable to new subjects, and were equally apparent in meaningful, social stimuli as in non-semantic pattern (fractal) images, indicating that lower-level features may be driving the effect [51, 52]. One of the most diagnostic features was the patients’ spatially restricted fixation patterns (Hedges *g* = 1.4, *p* < 10^–31^). This same pattern has been shown to occur to a lesser extent in bipolar disorder [52] and unaffected siblings [53]. In a more recent longitudinal study with a three year follow-up, unmedicated clinical high-risk subjects who went on to convert to a psychotic disorder (*n* = 21) had smaller saccade amplitudes during free viewing at baseline than those who did not convert (*n* = 87; *d* = 0.70) [54]. In that study, a logistic regression model could jointly distinguish the two groups of patients with high accuracy (AUC = 0.80, specificity = 0.84, sensitivity = 0.67). However, the study did not quantify fixation dispersion or overall scan path length, and thus may have underestimated the potential predictive utility of free viewing.

An advantage to a battery of visual tests is that the components will be only weakly correlated even when the tasks are highly similar [55]. Therefore, finding a group difference with one task will very often pick up variance different than that picked up by others; and combining such tasks will yield a more powerful diagnostic tool. In other words, even if no specific visual task generates clinically useful group differences on its own, a battery of such tasks could potentially do so. This contrasts with cognitive tasks, which are highly intercorrelated as captured by the “g” construct of general intelligence [56].

### Brain markers of abnormal vision in early psychosis

Resting-state functional hyperconnectivity between the thalamus and parts of occipital cortex (Fig. 2A–C) has been demonstrated in larger samples (163 HCs, 151 SZs) independently of illness duration; the group difference could not be explained by antipsychotic medication dose or scanner motion [57]. Such hyperconnectivity has been also shown by other investigators (90 HCs, 90 SZs) [58] and may be partly explained by weaker anatomical connections between these same areas [59]. Thalamo-cortical visual functional hyperconnectivity has also been documented in affective and non-affective psychosis patients within 5 years of their first psychotic episode, when compared to healthy controls (*n* = 32, 81, 52, respectively) [60]. Finally, CHR patients who converted to a psychotic disorder (*n* = 21) displayed more visual thalamo-cortical connectivity at baseline than those who did not convert (*n* = 222) (Fig. 2B) [61]. This group difference was prior to cluster-wise type I error correction but it did appear to overlap with the hyperconnected regions of the more chronic patients (Fig. 2A).

In scalp recorded visual evoked potential (VEP) studies, brief achromatic checker patterns have elicited a reduced P1 waveform amplitude in schizophrenia patients compared to healthy controls and major depression patients [62, 63]. The group differences were typically large (*d* ≥ 0.7) and independent of medication dose and illness duration [64]. The alterations could not be explained by poor attention since patients could often perform normally on an accompanying rapid object recognition task [65, 66] and since the group difference could emerge even when an eye-tracker ensured proper fixation [62]. Critically, P1 amplitude reductions have also been documented over occipital electrodes in FEPs (Fig. 2D) [65]. When the waveforms were measured from electrodes outside of the occipital cortex, group differences would diminish or disappear entirely, attesting to the visual nature of the effect [63, 67].

In the realm of oscillations, a simple visual change detection task during MEG has revealed high gamma power reductions (57–67 Hz) in FEPs (*n* = 26) relative to healthy controls (*n* = 49, *d* = 1.14, *p* < 0.01; Fig. 2E, F) [68]. Unmedicated FEPs (*n* = 15) have also exhibited reduced high gamma-band power (>60 Hz; *d* = 0.7–1.0) when attempting to recognize vague black-and-white pictures (so-called “Mooney faces”) [69], similar to what has been found in more chronic patients [70].

Structural MRI variables can be difficult to interpret [71]. Nevertheless, multiple small studies have reported reduced white matter volume or fractional anisotropy in the calcarine cortex (V1) and the frontal-occipital fasciculus during the transition to psychosis [72]. Fractional anisotropy of the latter structure is reduced in drug-naïve FEPs (25 patients, 51 HCs, *d* > 1.1 for each hemisphere) [73] and has been shown to correlate with backward masking severity in psychosis [74].

Other brain-based differences are worth briefly mentioning; these include more numerous interconnected (“hub”) visual regions in early-stage psychosis [75], more weakly interconnected visual areas across different illness durations [57, 68], and a more weakly interconnected whole-brain functional network that consists of many visual nodes and that is linked to high polygenic risk for psychosis [76].

### Retinal markers of early psychosis

**Fig. 3:**
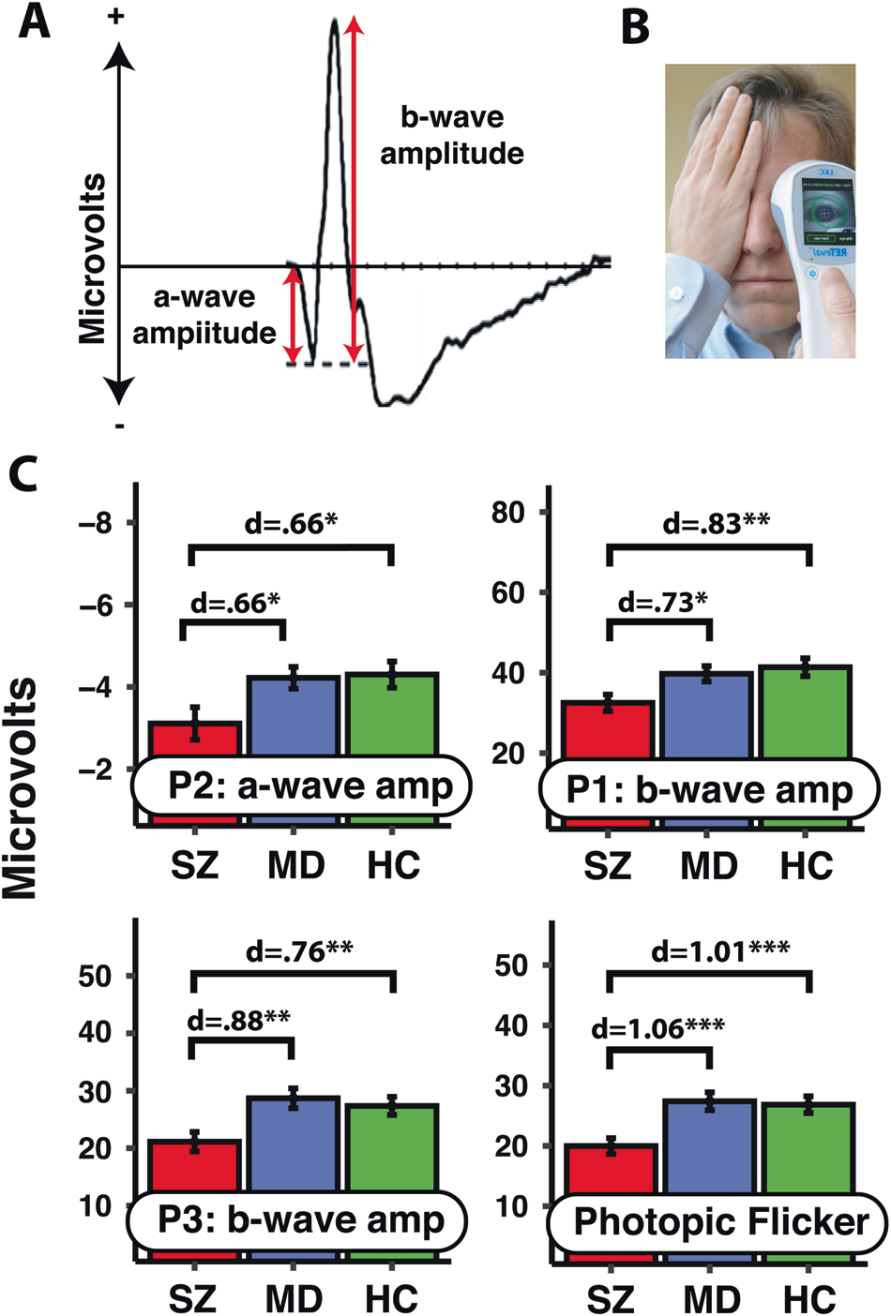
Electroretinographic markers of early psychosis. **A** Two example waveforms that have distinguished schizophrenia patients from healthy controls and mood disorder patients (photopic flicker response not shown). **B** Using a portable handheld device, light-adapted (photopic) retinal functioning of different cell types can be measured within 15 min. **C** Four ERG variables that have significantly distinguished schizophrenia patients from well-matched healthy controls and people with major depression (the most common final diagnosis among those at clinical high risk for psychosis; *n* = 25/group). The a- and b-wave amplitudes were measured under three photopic viewing conditions (P1, P2, P3); the photopic flicker response was measured under a fourth condition (for details see [81]). All effects were in a direction that could be anticipated from past research. *p < 0.05, **p < 0.01, ***p < 0.001.

Schizophrenia impacts N-methyl-D-aspartate (NMDA) receptors throughout the entire brain [9, 77], the retina and brain grow out of the same embryonic tissue, and NMDA receptor activity influences functioning of all major retinal cell types (amacrine, bipolar, photoreceptor, horizontal, ganglion) [78]. Therefore, it would be surprising if there were no retinal differences in schizophrenia. A major way to demonstrate such differences is through electroretinography (ERG), which detects retinally generated electrical activity in response to a flashing light stimulus. Depending on the stimulus properties, the resulting waveform can be decomposed into: an a-wave, reflecting hyperpolarization of photoreceptors; a b-wave, which derives from the depolarization of a complex of Müller and ON-center bipolar cells; and a photopic flicker response, which is defined by a series of positive peaks and is byproduct of cone functioning [79]. In a large study (*n* ≥ 150/group), when retinal responses were averaged across multiple luminance levels, lightness-adapted cone a- and b-wave amplitudes were smaller in schizophrenia relative to healthy controls (*d* = −0.49, −0.70); the b-wave latency was also prolonged (*d* = 1.29) [80]. The effects were highly replicable across 2-month durations. Critically, none of these effects depended on illness duration, at least not after controlling for age, suggesting that they may be apparent by the first psychotic episode. Furthermore, a stepwise logistic regression model could distinguish schizophrenia patients from a medication-matched subsample of bipolar patients via cone a-wave amplitude and latency (45/group; AUC = 0.83, sensitivity = 0.80, specificity = 0.82). Electroretinography conducted with a portable device has also distinguished schizophrenia patients from major depression patients for the a-wave amplitude, b-wave amplitude, and photopic flicker response (all *d* > 0.65; see Fig. 3C) [81].

### Visual testing in the clinic

The foregoing visual tests can often be administered in real-world clinical settings. The behavioral tests can be conducted with commercial “gaming” monitors (120 Hz refresh rate; <$1000), an ordinary desktop computer, and freely available software for stimulus presentation and screen gamma correction (e.g., PsychoPy [82]). Fixation dispersion can be measured with increasingly affordable eye-trackers (e.g., Gazepoint GP3; < $1000; 60 Hz, 0.5–1.0 deg precision) [83]. Retinal functioning can be assayed non-invasively with a portable, handheld FDA-approved “RETeval” device, which automatically adjusts for pupil size on a trial-by-trial basis in nondilated eyes [84]. The device is well tolerated and imposes few inclusion/exclusion criteria. The behavioral, oculomotor, and electroretinography tests can each be performed within 5–15 min and therefore can readily be appended to other assessments routinely administered in clinical high-risk clinics.

The brain-based data (e.g., resting-state, VEP, gamma oscillations) obviously require significant infrastructure and expertise to collect. However, VEP and gamma oscillation data can often be obtained in under one hour and such sessions are generally well tolerated with few contraindications. Moreover, fMRI-based resting-state group differences have emerged with different scanners, data types (multiband, single-band), and preprocessing strategies, and require minimal effort from the subject. It is true that resting-state connectivity metrics are prone to motion artifacts. However, with proper head padding and coaching, group-wise motion differences are small, especially when comparing psychosis patients to individuals with other forms of psychopathology [61, 85]. Moreover, new and more effective methods for controlling for motion are on the horizon, including multi-echo fMRI and prospective high-motion scan detection [86, 87].

### Mechanistic implications

It is beyond the scope of this work to delineate the physiological mechanisms engaged by each measure and how they relate to the emergence of psychosis. However, many of the measures have been extensively investigated in humans and other animals, in clinical and non-clinical populations, and with multiple methodologies (EEG, fMRI, TMS, single-cell recordings), indicating that their mechanisms for predicting psychosis could become readily known. For example, ketamine models suggest that NMDA receptor hypofunction may strengthen visual thalamo-cortical connectivity [88] and distort V1 activity during visual object recognition [89]. Contrast sensitivity tests might flag impending psychosis because they depend on dopamine synthesis [90], which itself predicts a transition to psychosis [91]. The ERG b-wave amplitude is generated by D_1_ receptor signaling on cone ON-bipolar and horizontal cells [90], implying again that alterations of this waveform could predict psychosis through its sensitivity to dopamine. Reduced gamma-band synchrony has been attributed to parvalbumin or somatostatin expressing GABAergic interneurons [68, 92, 93]. Therefore, not only could visual system assessments predict who will develop a psychotic disorder, they could also provide critical clues on the cell types, circuits, and neurotransmitter systems that underlie such a transition.

Note that visual measures may be clinically useful even if they are not yet mechanistically understood. For instance, the Hopkins Verbal Learning Test-Revised has improved psychosis risk estimates in CHR studies [22], yet it remains unclear why this is so or why other cognitive tests (e.g., the continuous performance or vocabulary tests) have not achieved a similar outcome [94]. Likewise, in the treatment domain, we now understand that antipsychotic medications modulate the dopamine D_2_ receptor but this discovery happened more than two decades after it was shown to ameliorate positive symptoms [95]. Therefore, demonstrating a test’s predictive power can precede and thereby motivate a more focused search for the mechanisms responsible, which in turn can provide novel targets for treatment and intervention.

### Addressing objections and limitations

One may object that psychosis alters each of the senses and thus any battery that focuses only on vision will be necessarily limited. This objection, while technically correct, overlooks the fact that the human cerebral cortex is devoted to more to vision than any other sense and that we know more about the visual system than any other sensory system, implying that behavioral results can have direct implications for our understanding of brain functioning. Moreover, demonstrating the utility of visual tests in no way precludes and may even serve to justify the development of tests based on other sense modalities. For example, Fig. 2B (showing thalamic hyperconnectivity with the somatomotor cortex) suggests that tests of motor and tactile functioning could constitute a novel and complementary family of predictive measures. Our argument therefore is not that vision should be used alone in screening, but alongside other promising tests to facilitate a timely, accurate clinical prediction.

A related objection is that most of the foregoing assessments were developed in the context of schizophrenia rather than psychosis more broadly, but psychosis risk calculators apply to affective psychosis as well [22]. Therefore, our tests may not be as useful in ordinary clinical contexts, where a transition to any psychotic disorder remains the question of interest. First, it is simply too soon to decide whether the above-described assessments are useless for flagging other forms of psychosis and, indeed, some have already differentiated individuals with affective early psychosis [60]. Second, if the above-mentioned tests primarily differentiate non-affective psychosis patients, then that itself would be important; it would demonstrate novel biological and behavioral differences between patient subtypes that could justify a search for more individualized treatments and interventions.

A more general objection is that it is more fruitful to examine functional outcome rather than “transition to psychosis”, which inherently assumes a case/control approach [96, 97]. However, people with psychotic disorders typically have worse functional outcomes than people who do not, at least in some domains [98]. Therefore, identifying people with psychosis is tantamount to identifying people with poor functional outcome. An analogous point applies to non-affective psychosis. A large longitudinal study of patients with affective and non-affective psychosis has shown that individuals with 10 days or more of psychosis outside of a mood episode at baseline had steeper declines in their Global Assessment in Functioning scores (up to 10 years later) than those who did not [99]. Thus, if the goal is to predict eventual changes in functioning, then this goal will largely be accomplished by identifying people who develop non-affective psychosis. Finally, many of the measures have been associated with *negative symptoms* in at least some studies; these include self-reported visual distortions [46], weaker collinear facilitation [46], stronger backward masking [38], spatially restricted eye movements [100], visual thalamo-cortical hyperconnectivity [101], attenuated P1 amplitude (VEP) [64], attenuated photopic ERG amplitude [102], and white matter microstructural abnormalities of the inferior fronto-occipital fasciculus [73]. Consequently, not only might abnormal vision predict poor outcome, it may also predict the negative symptoms that often accompany poor outcome and that often emerge first in illness development [103].

General limitations to our approach are worth noting. First, many visual tests will not be clinically predictive. For example, thalamic connectivity is normal for many visual regions (see Fig. 2A, B), backward masking deficits are more apparent for some stimulus configurations than for others [104], and free-viewing eye-tracking tasks better discriminate patients than smooth pursuit or steady fixation [51]. The reason why only some tests or brain regions discriminate psychosis patients remains an interesting open question. Visual testing will also likely not be useful for screening for psychosis in the general population. Bayesian analysis straightforwardly shows that—because psychosis is a low base-rate illness (~1% prevalence)—diagnostic tests with outstanding sensitivity and specificity will still yield unacceptably high false alarm rates [105].

### Future directions

The visual system is seldom viewed as a viable source of biomarkers in psychosis research [3, 106]. This is unfortunate because examples spanning all major levels of analysis—from phenomenology to behavior to brain and retinal functioning—have shown how visual system assessment could help identify individuals who go on to develop a psychotic disorder. Three markers have already longitudinally distinguished individuals with psychosis: visual basic symptoms, visual thalamo-cortical hyperconnectivity, and spatially restricted eye movements. The remaining markers have mostly been demonstrated in first-episode patients. All of the above assessments must be validated longitudinally in CHR populations alongside more established neurocognitive and clinical predictors. Just as only some cognitive deficits turned out to be uniquely predictive in CHR studies, so too will only a subset of visual abnormalities. This opinion piece is therefore a call for action—to harness visual tests to consider which might improve clinical prediction in CHR populations.

## References

[1] Insel TR (2010). Rethinking schizophrenia. Nature..

[2] Chong HY, Teoh SL, Wu DB-C, Kotirum S, Chiou C-F, Chaiyakunapruk N. Global economic burden of schizophrenia: a systematic review. Neuropsychiatr Dis Treat. 2016;12:357–73. 10.2147/NDT.S96649 PMC4762470 26937191

[3] Fusar-Poli P, Salazar de Pablo G, Correll CU, Meyer-Lindenberg A, Millan MJ, Borgwardt S (2020). Prevention of psychosis: advances in detection, prognosis, and intervention. JAMA Psychiatry.

[4] Thompson E, Millman ZB, Okuzawa N, Mittal V, Devylder J, Skadberg T (2015). Evidence-based early interventions for individuals at clinical high risk for psychosis: a review of treatment components. J Nerv Ment Dis.

[5] Nelson B, McGorry P (2020). The prodrome of psychotic disorders. Child Adolesc Psychiatr Clin N Am.

[6] Beck K, Andreou C, Studerus E, Heitz U, Ittig S, Leanza L (2019). Clinical and functional long-term outcome of patients at clinical high risk (CHR) for psychosis without transition to psychosis: a systematic review. Schizophr Res.

[7] Nicholl D, Akhras KS, Diels J, Schadrack J (2010). Burden of schizophrenia in recently diagnosed patients: Healthcare utilisation and cost perspective. Curr Med Res Opin.

[8] Van Essen DC. Organization of visual areas in macaque and human cerebral cortex. In: Chalupa L, Werner J, editors. The visual neurosciences, vol. 1, Cambridge, MA: MIT Press; 2003. p. 507–21.

[9] Trubetskoy V, Pardiñas AF, Qi T, Panagiotaropoulou G, Awasthi S, Bigdeli TB, et al. Mapping genomic loci implicates genes and synaptic biology in schizophrenia. Nature. 2022;604:502–08. 10.1038/s41586-022-04434-5 PMC9392466 35396580

[10] Owen MJ, Sawa A, Mortensen PB (2016). Schizophrenia. Lancet.

[11] Meng X, Iraji A, Fu Z, Kochunov P, Belger A, Ford J, et al. Multimodel order independent component analysis: a data-driven method for evaluating brain functional network connectivity within and between multiple spatial scales. Brain Connect. 2021. 10.1089/brain.2021.0079. 10.1089/brain.2021.0079 PMC9529308 34541879

[12] Waters F, Fernyhough C (2017). Hallucinations: a systematic review of points of similarity and difference across diagnostic classes. Schizophr Bull.

[13] Keane BP, Cruz LN, Paterno D, Silverstein SM (2018). Self-reported visual perceptual abnormalities are strongly associated with core clinical features in psychotic disorders. Front Psychiatry.

[14] Phillipson OT, Harris JP (1985). Perceptual changes in schizophrenia: a questionnaire survey. Psychol. Med.

[15] Huang J, Song X, Xu Y, Wang L, Li Y, Tian H (2020). Reliability and diagnostic validity of a novel visual disturbance subjective experience scale in chinese patients with schizophrenia. Psychiatry Clin Psychopharmacol.

[16] Waters F, Collerton D, ffytche DH, Jardri R, Pins D, Dudley R (2014). Visual hallucinations in the psychosis spectrum and comparative information from neurodegenerative disorders and eye disease. Schizophr Bull.

[17] Lin A, Wood SJ, Nelson B, Beavan A, McGorry P, Yung AR (2015). Outcomes of nontransitioned cases in a sample at ultra-high risk for psychosis. Am J Psychiatry.

[18] Salgado JF (2018). Transforming the area under the normal curve (AUC) into cohen’s d, pearson’s rpb, odds-ratio, and natural log odds-ratio: Two conversion tables. Eur J Psychol Appl Leg Context.

[19] Silverstein SM. Visual perception disturbances in schizophrenia: a unified model. In: Li M, Spaulding WD, editors. The neuropsychopathology of schizophrenia, vol. 63. 3rd ed. Cham: Springer International Publishing; 2016. p. 77–132. 10.1007/978-3-319-30596-7_4 27627825

[20] Silverstein SM, Keane BP (2011). Perceptual organization impairment in schizophrenia and associated brain mechanisms: review of research from 2005 to 2010. Schizophr Bull.

[21] Silverstein SM, Rosen R (2015). Schizophrenia research: cognition. Schizophr Res Cogn.

[22] Cannon TD, Yu C, Addington J, Bearden CE, Cadenhead KS, Cornblatt BA (2016). An individualized risk calculator for research in prodromal psychosis. Am J Psychiatry.

[23] Huber G, Gross G (1989). The concept of basic symptoms in schizophrenic and schizoaffective psychoses. Recent Prog Med.

[24] Mathalon DH, Ford JM (2012). Neurobiology of schizophrenia: search for the elusive correlation with symptoms. Front Hum Neurosci.

[25] Vollmer-Larsen A, Handest P, Parnas J (2007). Reliability of measuring anomalous experience: the Bonn Scale for the Assessment of Basic Symptoms. Psychopathology..

[26] Parnas J, Handest P, Saebye D, Jansson L (2003). Anomalies of subjective experience in schizophrenia and psychotic bipolar illness. Acta Psychiatr Scand.

[27] Klosterkötter J, Hellmich M, Steinmeyer EM, Schultze-Lutter F (2001). Diagnosing schizophrenia in the initial prodromal phase. Arch Gen Psychiatry.

[28] Kahn RS, Keefe RSE (2013). Schizophrenia is a cognitive illness. JAMA Psychiatry.

[29] Mollon J, David AS, Zammit S, Lewis G, Reichenberg A (2018). Course of cognitive development from infancy to early adulthood in the psychosis spectrum. JAMA Psychiatry.

[30] Kingdom FAA, Prins N. Psychophysics. 2nd ed. New York: Academic Press; 2016.

[31] Kiss I, Fábián Á, Benedek G, Kéri S (2010). When doors of perception open: Visual contrast sensitivity in never-medicated, first-episode schizophrenia. J Abnorm Psychol.

[32] Kelemen O, Kiss I, Benedek G, Kéri S (2013). Perceptual and cognitive effects of antipsychotics in first-episode schizophrenia: The potential impact of GABA concentration in the visual cortex. Prog Neuropsychopharmacol Biol Psychiatry.

[33] Chen Y, Levy DL, Sheremata S, Nakayama K, Matthysse S, Holzman PS (2003). Effects of typical, atypical, and no antipsychotic drugs on visual contrast detection in schizophrenia. Am J Psychiatry.

[34] Schechter I, Butler PD, Jalbrzikowski M, Pasternak R, Saperstein AM, Javitt DC (2006). A new dimension of sensory dysfunction: stereopsis deficits in schizophrenia. Biol Psychiatry.

[35] Wang Z, Yu Z, Pan Z, Zhao K, Zhao Q, Zhou D (2018). Impaired binocular depth perception in first-episode drug-naive patients with schizophrenia. Front Psychol.

[36] Hui L, Xia Sen H, Shu Tang A, Feng Zhou Y, Zhong Yin G, Long Hu X (2017). Stereopsis deficits in patients with schizophrenia in a Han Chinese population. Sci Rep.

[37] Sponheim SR, Sass SM, Noukki AL, Hegeman BM (2013). Fragile early visual percepts mark genetic liability specific to schizophrenia. Schizophr Bull.

[38] Green MF, Lee J, Wynn JK, Mathis KI (2011). Visual masking in schizophrenia: overview and theoretical implications. Schizophr Bull.

[39] Pukrop R, Schultze-Lutter F, Ruhrmann S, Brockhaus-Dumke A, Tendolkar I, Bechdolf A (2006). Neurocognitive functioning in subjects at risk for a first episode of psychosis compared with first- and multiple-episode schizophrenia. J Clin Exp Neuropsychol.

[40] Herzog MH, Kopmann S, Brand A (2004). Intact figure-ground segmentation in schizophrenia. Psychiatry Res.

[41] Favrod O, da Cruz JR, Roinishvili M, Berdzenishvili E, Brand A, Figueiredo P (2019). Electrophysiological correlates of visual backward masking in patients with major depressive disorder. Psychiatry Res Neuroimaging.

[42] Favrod O, Roinishvili M, da Cruz JR, Brand A, Okruashvili M, Gamkrelidze T (2018). Electrophysiological correlates of visual backward masking in patients with first episode psychosis. Psychiatry Res Neuroimaging.

[43] Polat U, Sagi D (1994). Spatial interactions in human vision: from near to far via experience-dependent cascades of connections. Proc Natl Acad Sci USA.

[44] Must A, Janka Z, Benedek G, Kéri S (2004). Reduced facilitation effect of collinear flankers on contrast detection reveals impaired lateral connectivity in the visual cortex of schizophrenia patients. Neurosci Lett.

[45] Kéri S, Kelemen O, Benedek G, Janka Z (2005). Lateral interactions in the visual cortex of patients with schizophrenia and bipolar disorder. Psychological Med.

[46] Kéri S, Kiss I, Kelemen O, Benedek G, Janka Z (2005). Anomalous visual experiences, negative symptoms, perceptual organization and the magnocellular pathway in schizophrenia: a shared construct?. Psychol Med.

[47] Keane BP, Paterno D, Crespo LP, Kastner S, Silverstein SM (2019). Smaller visual arrays are harder to integrate in schizophrenia: Evidence for impaired lateral connections in early vision. Psychiatry Res.

[48] Keane BP, Paterno D, Kastner S, Krekelberg B, Silverstein SM (2019). Intact illusory contour formation but equivalently impaired visual shape completion in first- and later-episode schizophrenia. J Abnorm Psychol.

[49] Keane BP, Erlikhman G, Serody MR, Silverstein SM (2021). A brief psychometric test reveals robust shape completion deficits in schizophrenia that are less severe in bipolar disorder. Schizophr Res.

[50] Keane BP, Paterno D, Kastner S, Silverstein SM (2016). Visual integration dysfunction in schizophrenia arises by the first psychotic episode and worsens with illness duration. J Abnorm Psychol.

[51] Benson PJ, Beedie SA, Shephard E, Giegling I, Rujescu D, St Clair D (2012). Simple viewing tests can detect eye movement abnormalities that distinguish schizophrenia cases from controls with exceptional accuracy. Biol Psychiatry.

[52] Bestelmeyer PEG, Tatler BW, Phillips LH, Fraser G, Benson PJ, St Clair D (2006). Global visual scanning abnormalities in schizophrenia and bipolar disorder. Schizophr Res.

[53] Takahashi S, Tanabe E, Yara K, Matsuura M, Matsushima E, Kojima T (2008). Impairment of exploratory eye movement in schizophrenia patients and their siblings. Psychiatry Clin Neurosci.

[54] Zhang D, Xu L, Xie Y, Tang X, Hu Y, Liu X, et al. Eye movement indices as predictors of conversion to psychosis in individuals at clinical high risk. Eur Arch Psychiatry Clin Neurosci. 2022:1–11. 10.1007/s00406-022-01463-z 35857090

[55] Grzeczkowski L, Clarke AM, Francis G, Mast FW, Herzog MH (2017). About individual differences in vision. Vis Res.

[56] Gläscher J, Rudrauf D, Colom R, Paul LK, Tranel D, Damasio H (2010). Distributed neural system for general intelligence revealed by lesion mapping. Proc Natl Acad Sci USA.

[57] Damaraju E, Allen EA, Belger A, Ford JM, McEwen S, Mathalon DH (2014). Dynamic functional connectivity analysis reveals transient states of dysconnectivity in schizophrenia. Neuroimage Clin.

[58] Anticevic A, Cole MW, Repovs G, Murray JD, Brumbaugh MS, Winkler AM (2014). Characterizing thalamo-cortical disturbances in schizophrenia and bipolar illness. Cereb Cortex.

[59] Giraldo-Chica M, Rogers BP, Damon SM, Landman BA, Woodward ND (2018). Prefrontal-thalamic anatomical connectivity and executive cognitive function in schizophrenia. Biol Psychiatry.

[60] Fu Z, Iraji A, Sui J, Calhoun VD (2021). Whole-brain functional network connectivity abnormalities in affective and non-affective early phase psychosis. Front Neurosci.

[61] Anticevic A, Haut K, Murray JD, Repovs G, Yang GJ, Diehl C (2015). Association of thalamic dysconnectivity and conversion to psychosis in youth and young adults at elevated clinical risk. JAMA Psychiatry.

[62] Bedwell JS, Spencer CC, Chan CC, Butler PD, Sehatpour P, Schmidt J (2018). The P1 visual-evoked potential, red light, and transdiagnostic psychiatric symptoms. Brain Res.

[63] Yeap S, Kelly SP, Sehatpour P, Magno E, Javitt DC, Garavan H (2006). Early visual sensory deficits as endophenotypes for schizophrenia: high-density electrical mapping in clinically unaffected first-degree relatives. Arch Gen Psychiatry.

[64] Yeap S, Kelly SP, Sehatpour P, Magno E, Garavan H, Thakore JH (2008). Visual sensory processing deficits in Schizophrenia and their relationship to disease state. Eur Arch Psychiatry Clin Neurosci.

[65] Yeap S, Kelly SP, Thakore JH, Foxe JJ (2008). Visual sensory processing deficits in first-episode patients with Schizophrenia. Schizophr Res.

[66] Lalor EC, Sanctis PD, Krakowski MI, Foxe JJ (2012). Visual sensory processing deficits in schizophrenia: Is there anything to the magnocellular account?. Schizophr Res.

[67] Katsanis J, Iacono WG, Beiser M (1996). Visual event-related potentials in first-episode psychotic patients and their relatives. Psychophysiology..

[68] Grent-‘t-Jong T, Gajwani R, Gross J, Gumley AI, Krishnadas R, Lawrie SM, et al. Association of magnetoencephalographically measured high-frequency oscillations in visual cortex with circuit dysfunctions in local and large-scale networks during emerging psychosis. JAMA Psychiatry. 2020. 10.1001/jamapsychiatry.2020.0284. 10.1001/jamapsychiatry.2020.0284 PMC7097849 32211834

[69] Sun L, Castellanos N, Grützner C, Koethe D, Rivolta D, Wibral M (2013). Evidence for dysregulated high-frequency oscillations during sensory processing in medication-naïve, first episode schizophrenia. Schizophr Res.

[70] Grützner C, Wibral M, Sun L, Rivolta D, Singer W, Maurer K (2013). Deficits in high- (>60 Hz) gamma-band oscillations during visual processing in schizophrenia. Front Hum Neurosci.

[71] Weinberger DR, Radulescu E (2020). Structural magnetic resonance imaging all over again. JAMA Psychiatry.

[72] Merritt K, Luque Laguna P, Irfan A, David AS (2021). Longitudinal structural MRI findings in individuals at genetic and clinical high risk for psychosis: a systematic review. Front Psychiatry.

[73] Serpa MH, Doshi J, Erus G, Chaim-Avancini TM, Cavallet M, van de Bilt MT (2017). State-dependent microstructural white matter changes in drug-naïve patients with first-episode psychosis. Psychol. Med.

[74] Berkovitch L, Charles L, Del Cul A, Hamdani N, Delavest M, Sarrazin S (2021). Disruption of conscious access in psychosis is associated with altered structural brain connectivity. J Neurosci.

[75] Hummer TA, Yung MG, Goñi J, Conroy SK, Francis MM, Mehdiyoun NF (2020). Functional network connectivity in early-stage schizophrenia. Schizophr Res.

[76] Cao H, Zhou H, Cannon TD (2020). Functional connectome-wide associations of schizophrenia polygenic risk. Mol Psychiatry.

[77] Braun U, Schäfer A, Bassett DS, Rausch F, Schweiger JI, Bilek E (2016). Dynamic brain network reconfiguration as a potential schizophrenia genetic risk mechanism modulated by NMDA receptor function. Proc Natl Acad Sci USA.

[78] Shen Y, Liu X-L, Yang X-L (2006). N-methyl-D-aspartate receptors in the retina. Mol Neurobiol.

[79] Young B, Eggenberger E, Kaufman D (2012). Current electrophysiology in ophthalmology. Curr Opin Ophthalmol.

[80] Hébert M, Mérette C, Gagné A-M, Paccalet T, Moreau I, Lavoie J (2020). The electroretinogram may differentiate schizophrenia from bipolar disorder. Biol Psychiatry.

[81] Demmin DL, Netser R, Roché MW, Thompson JL, Silverstein SM (2020). People with current major depression resemble healthy controls on flash Electroretinogram indices associated with impairment in people with stabilized schizophrenia. Schizophr Res.

[82] Peirce J, Gray JR, Simpson S, MacAskill M, Höchenberger R, Sogo H (2019). PsychoPy2: experiments in behavior made easy. Behav Res Methods.

[83] Brand J, Diamond SG, Thomas N, Gilbert-Diamond D (2021). Evaluating the data quality of the Gazepoint GP3 low-cost eye tracker when used independently by study participants. Behav Res Methods.

[84] Kato K, Kondo M, Sugimoto M, Ikesugi K, Matsubara H (2015). Effect of pupil size on flicker ERGs recorded with RET evalSystem: new mydriasis-free full-field ERG system. Investig Ophthalmol Vis Sci.

[85] Huang CC, Luo Q, Palaniyappan L, Yang AC, Hung CC, Chou KH (2020). Transdiagnostic and illness-specific functional dysconnectivity across schizophrenia, bipolar disorder, and major depressive disorder. Biol Psychiatry Cogn Neurosci Neuroimaging.

[86] Power JD, Plitt M, Gotts SJ, Kundu P, Voon V, Bandettini PA (2018). Ridding fMRI data of motion-related influences: Removal of signals with distinct spatial and physical bases in multiecho data. Proc Natl Acad Sci USA.

[87] Dosenbach NUF, Koller JM, Earl EA, Miranda-Dominguez O, Klein RL, Van AN (2017). Real-time motion analytics during brain MRI improve data quality and reduce costs. Neuroimage.

[88] Abram SV, Roach BJ, Fryer SL, Calhoun VD, Preda A, Erp TGM, et al. Validation of ketamine as a pharmacological model of thalamic dysconnectivity across the illness course of schizophrenia. Mol Psychiatry. 2022;27:2448–56. 10.1038/s41380-022-01502-0 PMC9135621 35422467

[89] van Loon AM, Fahrenfort JJ, van der Velde B, Lirk PB, Vulink NCC, Hollmann MW, et al. NMDA receptor antagonist ketamine distorts object recognition by reducing feedback to early visual cortex. Cereb Cortex. 2016;26:1986–96. 10.1093/cercor/bhv018 25662715

[90] Jackson CR, Ruan G-X, Aseem F, Abey J, Gamble K, Stanwood G (2012). Retinal dopamine mediates multiple dimensions of light-adapted vision. J Neurosci.

[91] Howes O, McCutcheon R, Stone J (2015). Glutamate and dopamine in schizophrenia: an update for the 21st century. J Psychopharmacol.

[92] Sohal VS, Zhang F, Yizhar O, Deisseroth K (2009). Parvalbumin neurons and gamma rhythms enhance cortical circuit performance. Nature..

[93] Veit J, Hakim R, Jadi MP, Sejnowski TJ, Adesnik H (2017). Cortical gamma band synchronization through somatostatin interneurons. Nat Neurosci.

[94] Seidman LJ, Shapiro DI, Stone WS, Woodberry KA, Ronzio A, Cornblatt BA (2016). Association of neurocognition with transition to psychosis: baseline functioning in the second phase of the North American Prodrome Longitudinal Study. JAMA Psychiatry.

[95] Kapur S, Mamo D (2003). Half a century of antipsychotics and still a central role for dopamine D_2_ receptors. Prog Neuropsychopharmacol Biol Psychiatry.

[96] van Os J, Guloksuz S (2017). A critique of the “ultra-high risk” and “transition” paradigm. World Psychiatry.

[97] Gold JM, Millman ZB, Dickinson D (2021). Enhancing prediction of psychosis risk with cognitive measures: how do we get to there from here?. JAMA Psychiatry.

[98] Carrión RE, McLaughlin D, Goldberg TE, Auther AM, Olsen RH, Olvet DM (2013). Prediction of functional outcome in individuals at clinical high risk for psychosis. JAMA Psychiatry.

[99] Kotov R, Leong SH, Mojtabai R, Erlanger ACE, Fochtmann LJ, Constantino E (2013). Boundaries of schizoaffective disorder: Revisiting Kraepelin. JAMA Psychiatry.

[100] Kojima T, Matsushima E, Nakajima K, Shiraishi H, Ando K, Ando H (1990). Eye movements in acute, chronic, and remitted schizophrenics. Biol Psychiatry.

[101] Chen M-H, Chang W-C, Bai Y-M, Huang K-L, Tu P-C, Su T-P (2020). Cortico-thalamic dysconnection in early-stage schizophrenia: a functional connectivity magnetic resonance imaging study. Eur Arch Psychiatry Clin Neurosci.

[102] Demmin DL, Davis Q, Roché M, Silverstein SM (2018). Electroretinographic anomalies in schizophrenia. J Abnorm Psychol.

[103] Correll CU, Schooler NR (2020). Negative symptoms in schizophrenia: a review and clinical guide for recognition, assessment, and treatment. Neuropsychiatr Dis Treat.

[104] Perez VB, Shafer KM, Cadenhead KS (2012). Visual information processing dysfunction across the developmental course of early psychosis. Psychological Med.

[105] Poldrack RA, Huckins G, Varoquaux G (2020). Establishment of best practices for evidence for prediction. JAMA Psychiatry.

[106] Lieberman JA, Small SA, Girgis RR (2019). Early detection and preventive intervention in schizophrenia: from fantasy to reality. Am J Psychiatry.

